# A protocol for metabolic characterization of human induced pluripotent stem cell-derived cardiomyocytes (iPS-CM)

**DOI:** 10.1016/j.mex.2019.05.028

**Published:** 2019-05-29

**Authors:** Alisha House, Erica Fatica, Rohan Shah, Jared Stergar, Ryan Pearce, Yana Sandlers

**Affiliations:** Department of Chemistry, Cleveland State University, Cleveland, OH 44115, United States

**Keywords:** Induced pluripotent stem cell-derived cardiomyocytes, Metabolic characterization, GCMS, LC–MS/MS

## Abstract

Recent advances in human induced pluripotent stem cell-derived cardiomyocytes (iPSCM) field offer a novel platform for modeling cardiac metabolism, heart diseases drug candidates screening and cardiac toxicity assessments. These workflows require a fully functional characterization of iPSCMs. Here we report a step by step protocol for iPSCM metabolic characterization. The described assays cover analysis of small metabolites involved in a vital metabolic pathways.

## Graphical abstract

**Figure:**
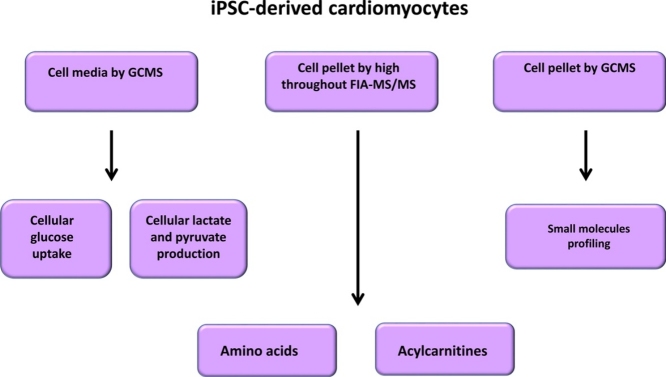

**Specifications Table**

**Table:** 

Subject Area:	*Biochemistry, Genetics and Molecular Biology*
More specific subject area:	*Metabolic profiling*
Protocol name:	*IPS-CM functional characterization*
Reagents/tools:	*Included in each section of the protocol*
Experimental design:	*Functional and beating induced pluripotent stem cells derived cardiomyocytes are cultured in a standard conditions or in the presence of 13C labeled glucose. Cells are harvested through the developed protocol, followed by mass spectrometry assays to analyze small metabolites, glucose uptake and lactate/pyruvate production*.
Trial registration:	*N/A*
Ethics:	*N/A*

**Value of the Protocol**

**Table:** 

• *new metabolic assays expand iPSCM functional analysis beyond already established techniques* • *assays can be applied to compare profiles, glucose uptake and lactate/pyruvate production in wild type vs. mutant cells* • *assays can be applied to monitor vital metabolites alterations during therapeutic interventions*

## Description of protocol

The discovery of cell reprogramming methods to obtain human induced pluripotent stem cells (iPSc) from adult somatic cells, followed by differentiation to cardiomyocytes, provides a unique opportunity to generate an unlimited number of induced pluripotent derives cardiomyocytes (iPS-CM) carrying specific cardiac genotypes and phenotypes. This invaluable model is an innovative tool for creating *in vitro* cardiac cellular models that can be utilized for new therapeutic assessments, screening of drug’s induced cardiac toxic effects and a discovery of as-yet unknown metabolic mechanisms that underlie cardiac manifestation of the inherited heart diseases. The ability to successfully translate cell-based studies into a clinical studies requires efficient tools for functional characterization of iPSC-CM. For this purpose electrophysiological and imaging techniques are utilized. These methods allow to monitor ion channel activities [1], measure action potentials, changes in Ca^+2^ fluxes [2], mitochondria viability and apoptosis [3]. Here we report new metabolic assays that allow to expand functional analysis beyond already established techniques and provide metabolic characteristics of iPSCM for the purpose of disease modeling, drug candidates screening and therapeutic agents cardiac safety assessments.

## Method details

### Differentiation

**Fig. 1:**
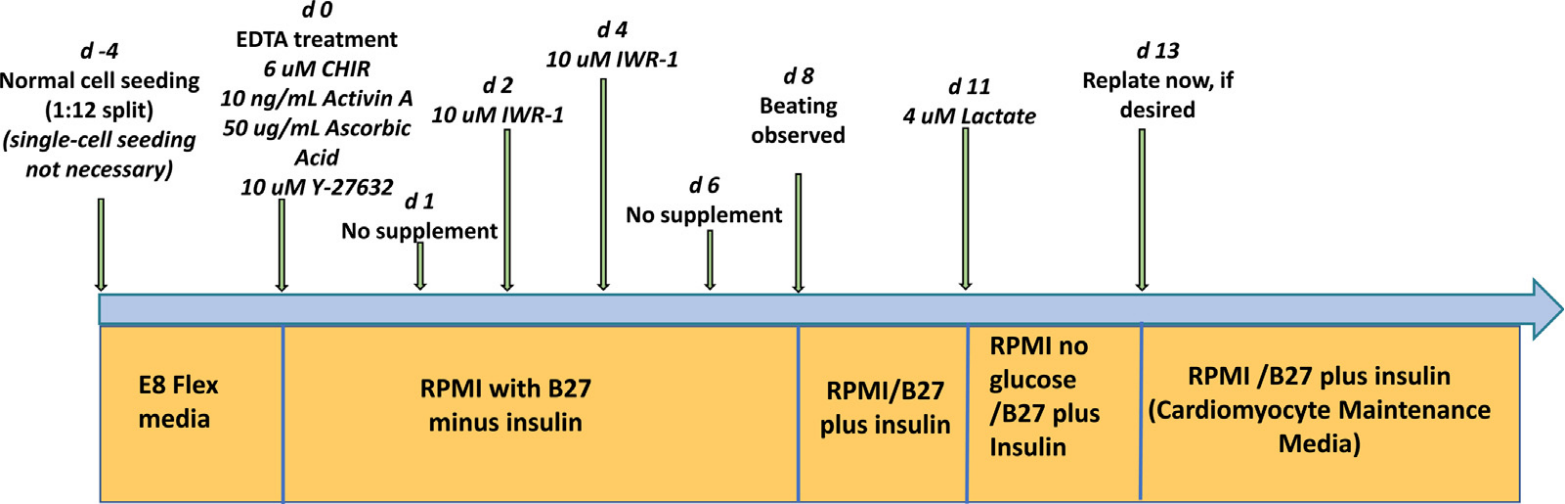
Differentiation protocol.

**Fig. 2:**
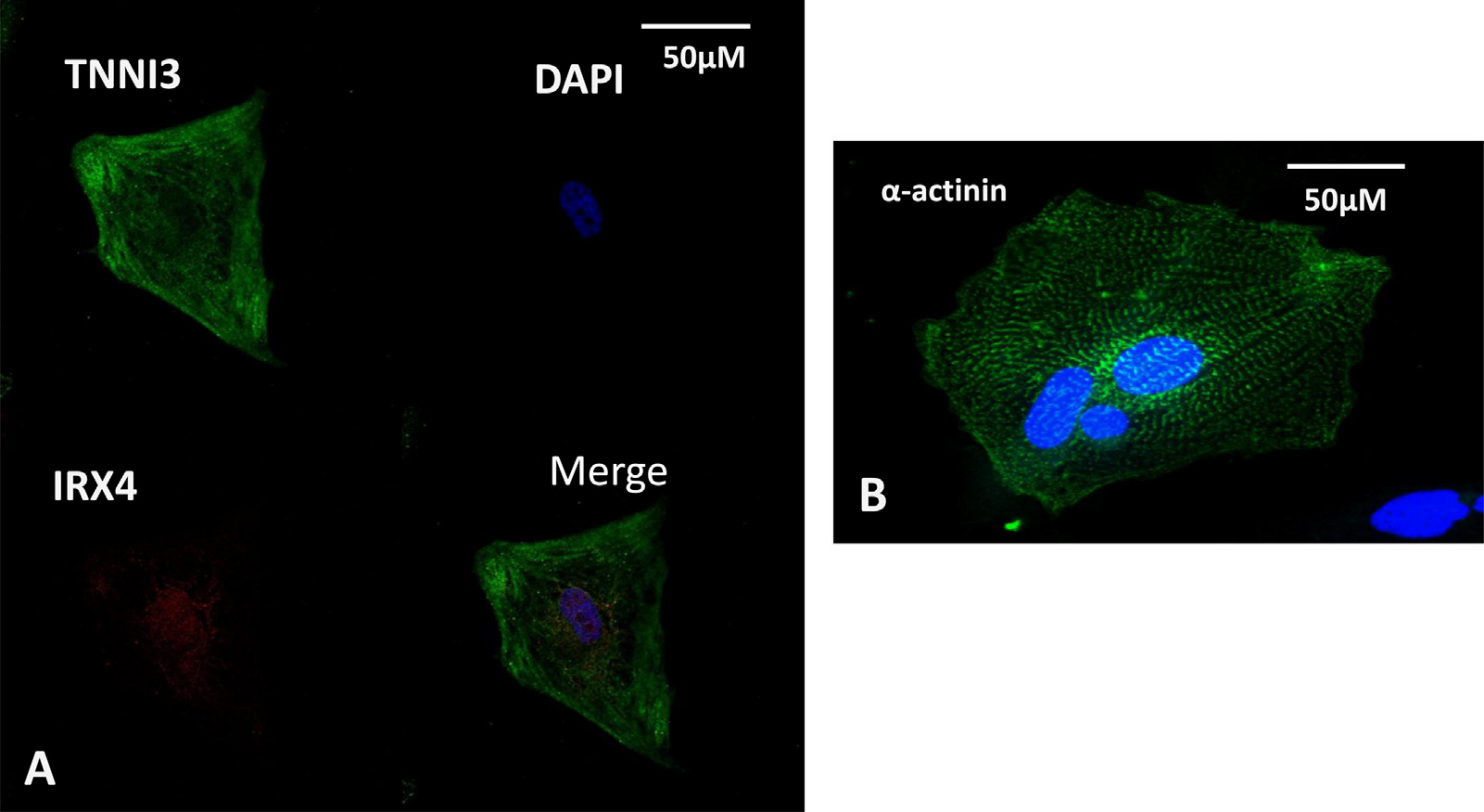
Immunostaining analysis for cardiac specific markers 42 days post differentiation. (A) Cardiac troponin I (green) and iroquois-class homeodomain protein (IRX4, red) and (B) α-actinin. Nuclei are shown by DAPI (blue).

iPSC cells were kindly contributed by Duke University core facility. Differentiation to cardiomyocytes was induced by the modified small molecules protocol [4] (Fig. 1). Briefly hiPSCs (passage >20) were passed at a 1:12 ratio and cultured on matrigel with Essential 8 Flex medium (Thermo Fisher Scientific) until ˜100% confluency. At Day 0, prior to medium change, E8 media was aspirated from cells, and they were washed with D-PBS. 1 mL of 0.5 mM EDTA in DPBS was added to each culture well. Plates were placed in incubator for approximately 35 s. Upon removal from incubator, dishes were gently tapped to dislodge only a small amount of cells. EDTA solution was aspirated, and differentiation medium was added directly to the EDTA-treated cells. The medium was changed to RPMI 1640 with B27 minus insulin supplement (A1895601, Life Technologies) with 6 μM CHIR99021,10 ng/mL Activin A, and 50 ug/mL ascorbic acid. On day 1, medium was changed to RPMI 1640 with B27 minus insulin supplement (referred to as basal differentiation supplement). On day 2, medium was changed to basal differentiation supplement and 10 μM IWR-1. On day 4, medium was refreshed with basal supplement and 10 μM IWR-1. Medium was changed on Day 6 to basal differentiation supplement (without IWR-1). On Day 8, and every other day thereafter, medium was refreshed with RPMI 1640 with B27 supplement (referred to as cardiomyocyte maintenance media). Spontaneously contracting cardiomyocytes (Fig. 2, Supplemental video) were first observed on day 9. Approximately 2 days after observing initial cardiomyocyte contractions, medium was changed to RPMI 1640 with no glucose and supplemented with 4 mM lactate. Medium was changed 72 h later to cardiomyocyte maintenance media.

### iPSCM harvest and extraction

All protocols below are adjusted for 1 well of a 6-well culture plate.

#### Materials

Labeled amino acids mixture, (Cambridge isotopes laboratories cat.no NSK-A)

Labeled acylcarnitines mixture, (Cambridge isotopes laboratories, cat.no NSK-B)

Tricarballylic acid (Sigma-Aldrich cat.no T53503) prepare 1 mM in water

Acetonitrile (VWR, cat.no BDH83640.100E LC/MS grade)

Chloroform (VWR, cat.no BDH83627 HPLC grade)

Water (VWR, cat.no BDH23595.100E HPLC grade)

Phosphate Buffered Saline (PBS), (VWR, cat.no 97063-658)

#### Metabolism quenching protocol

1. Aspirate culture media from iPSCM.
2. Wash cells twice with 1 mL cold PBS and one time with water. Ensure that water remains on cells for no more than 30 s.
3. Add 1 mL cold acetonitrile and incubate cells in −20 °C for 20 min. At the end of the 20 min period white protein precipitate will appear on culture dish bottom.
4. Add 0.75 mL of cold water and with the plastic scraper, scrape cells from the bottom of the culture dish.
5. Using a P1000 pipette, remove cell lysate to a 10 mL centrifuge tube.
6. Repeat steps 3–5 one more time. To ensures complete recovery of lysate it is recommended to use same cells scraper and pipette tip.
7. Add internal standard. Internal standards preparation and amounts varies with the analysis type. See notes #1-3.
8. To the collected cell lysate (step#4), add 1 mL of cold chloroform.
9. Vortex sample and centrifuge at 5000 rpm for 15 min. After completion of the centrifugation step, a clear separation will be observed between the polar (upper) phase, non-polar (lower) phase, and the inter-phase pellet that contains proteins and DNA.
10. From the upper polar phase, take an aliquot of 250 μL for acylcarnitines and amino acids assay and transfer to the clean tube. Label " amino acids and acylcarnitines".
11. Carefully remove and combine the rest of the upper polar phase with the bottom non-polar phase in a clean tube. Label "small cellular metabolites by GC/MS".
12. Add 50 μL methanol to the remaining protein pellet and evaporate solvent under nitrogen stream in room temperature.
13. Reconstitute protein with buffer and perform protein analysis by protein assay such as Bradford or bicinchoninic acid (BCA).

**Note #1**- Preparation of working solution NSK-A. Internal standard vial contains labeled standards for amino acids. For the stock solution, reconstitute dry powder in vial with 2 mL of water:methanol (1:1 v%/v%). Complete reconstitution of the contents of one vial in 2 mL produce stock solution of following metabolites. Alanine (2,3,3,3-^2^H_4_) 250 nmol/mL, L-Phenylalanine (ring-^13^C_6_) 250 nmol/mL, L-Leucine (5,5,5-^2^H_3_) 250 nmol/mL, L-Valine (^2^H_8_) 250 nmol/mL, L-Arginine: HCl (5-^13^C; 4,4,5,5-^2^H4) 250 nmol/L, L-Citrulline (5,5-^2^H_2_) 250 nmol/mL, L-Tyrosine (ring-^13^C_6_) 250 nmol/mL, L-Ornithine: HCl (5,5-^2^H_2_) 250 nmol/mL, L-Methionine (methyl-^2^H_3_) 250 nmol/mL, DL-Glutamic Acid (2,4,4-^2^H_3_) 250 nmol/mL, L-Aspartic Acid (2,3,3-^2^H_3_) 250 nmol/mL, Glycine (2-^13^C; ^15^N) 500 nmol/mL. For the working solution-dilute stock solution 1:10 in methanol. Add 50 μL of the working solution to the cell lysate.

**Note #2-** Preparation of working solution NSK-B. Internal standard vial contains labeled acylcarnitines standards. For the stock solution, reconstitute dry powder in vial with 2 mL of methanol. Complete reconstitution of the contents of one vial in 2 mL produce stock solution of following metabolites: Free carnitine (76 nmol/mL), ^2^H_3_-Acetylcarnitine (19 nmol/mL), ^2^H_3_-Propionylcarnitine (3.8 nmol/mL), ^2^H_3_-Butyrylcarnitine (3.8 nmol/mL), ^2^H_9_-Isovalerylcarnitine (3.8 nmol/mL), ^2^H_3_-Octanoylcarnitine (3.8 nmol/mL), ^2^H_9_-Myristoylcarnitine (3.8 nmol/mL), ^2^H_3_-Palmitoylcarnitine (7.6 nmol/mL). For the working solution-dilute stock solution 1:10^6^ in methanol. Add 100 μL of the working solution to the cell lysate.

**Note#3**- For the GCMS metabolic profile, add 25 μL of 1 mM tricarballylic acid

### Acylcarnitines and amino acids assay

#### Materials

3N HCl n-Butanol (Sigma-Aldrich, cat.no 87472)

Prepare mobile phase A for chromatographic separation: 20% Water (0.1% Formic acid): 80% Acetonitrile (0.1% Formic acid)

LC inline filter (VWR cat.no 97013-134)

#### Protocol

1. Dry 250 μL aliquots of the polar phase collected at step #10 and labeled "" *amino acids and acylcarnitines*" with internal standards (note #1 and #2).
2. To the dried sample add 60 μL HCl*-n-*Butanol, cap tubes and incubate at 65 °C for 30 min.
3. Cool to the room temperature and dry again.
4. Reconstitute with 100 μL of mobile phase A and transfer to vials for the analysis.

#### HPLC parameters

Introduce derivatized samples directly by the injection to the mass spectrometer instrument through the inline filter with no chromatographic separation. Keep inline filter in HPLC oven at 32 °C. Inject 7 μL of a sample into a flowing solvent at 50 μL/min flow rate for the total 1.8 run time. Use only mobile phase A isocratically.

#### MS/MS-tandem mass spectrometer

Use following tandem mass spectrometry scans for the high throughput: for the acylcarnitines precursor ion scan at *m/z* 85 (Table 1, only saturated C2-18 acylcarnitines are included), for amino acids neutral loss (NL 102, NL 119), and single reaction monitoring SRM *m/z* 231.1-*m/z* 70.1 for arginine (Table 2).

**Table 1:** Saturated acylcarnitines by acyl chain length as butyl esters and their molecular ions.

Chain length	[M+H]^+^/[M+H]^+^ Internal standard as butylesters
C2	260/263
C3	274/277
C4	288/299
C5	302/311
C6	316
C8	344/347
C10	372
C12	400
C14	428/437
C16	456/459
C18	484

**Table 2:** Amino acids as butyl esters and their molecular ions. Additional amino acids can be detected and quantified with the matching stable-isotopic labeled standards.

Target amino acid	[M+H]^+^/[M+H]^+^ Internal standard as butylesters
Alanine	146/150
Arginine	231/236
Aspartic Acid	246/249
Citrulline	232/234
Glutamic acid	260/263
Glycine	132/134
Leucine/Isoleucine	188/191
Methionine	206/209
Ornithine	189/191
Phenylalanine	222/228
Tyrosine	238/244
Valine	174/182

#### Data analysis

Perform data analysis by Chemoview 2.2 software (SCIEX) or comparable software. Calculate peak areas at 50% peak height.

#### Representative data

See Fig. 3, Fig. 4.

**Fig. 3:**
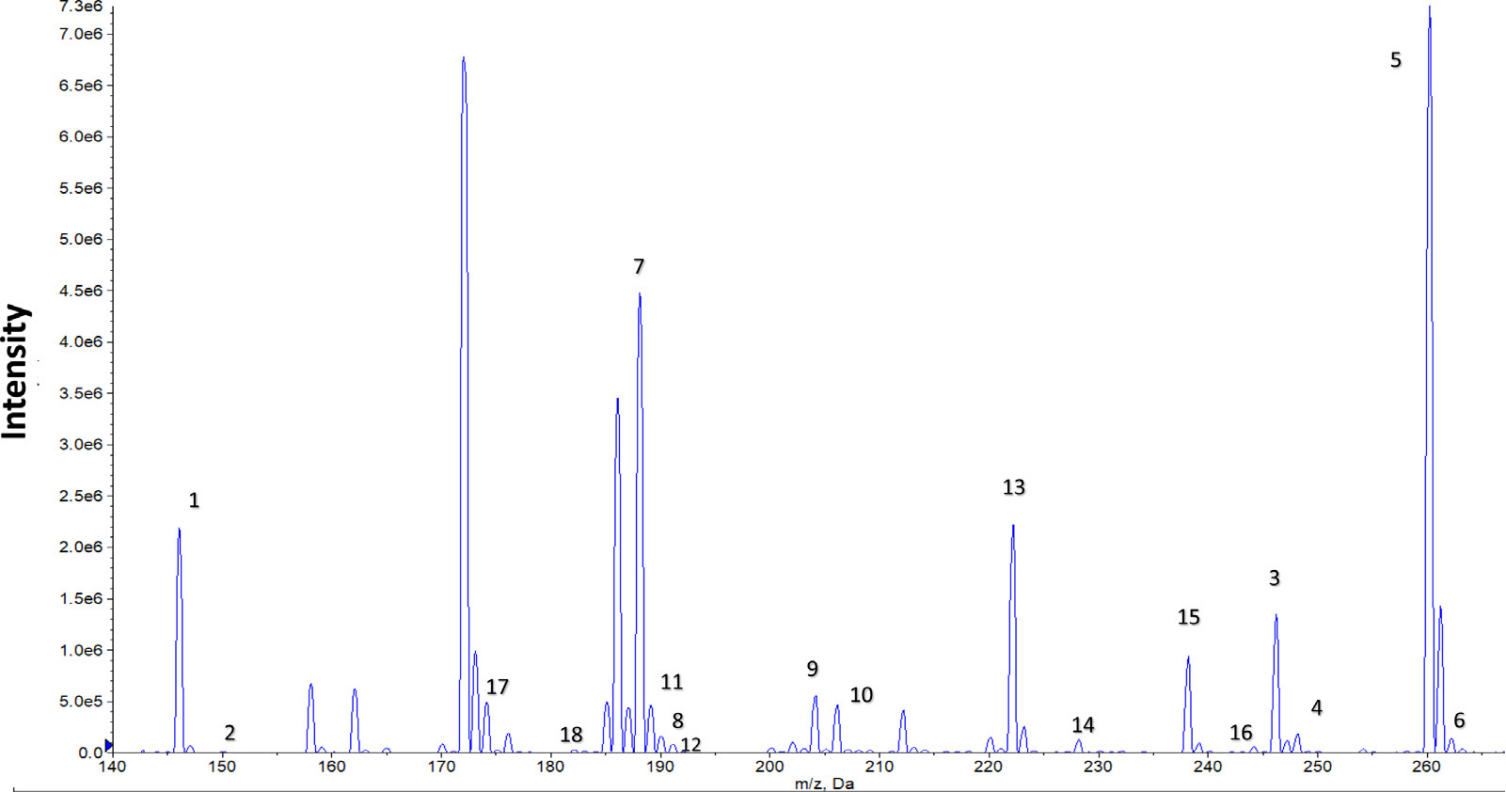
Amino acids analysis: representative *m/z* profile of NL 102 scan (1) alanine, (2) alanine ISTD, (3) aspartic acid, (4) aspartic acid ISTD, (5) glutamic acid (6) glutamic acid ISTD, (7) leucine/isoleucine, (8) leucine/isoleucine, (9) methionine, (10) methionine ISTD, (11) ornithine, (12) ornithine ISTD, (13) phenylalanine, (14) phenylalanine ISTD, (15) tyrosine, (16) tyrosine ISTD, (17) valine, (17) valine ISTD.

**Fig. 4:**
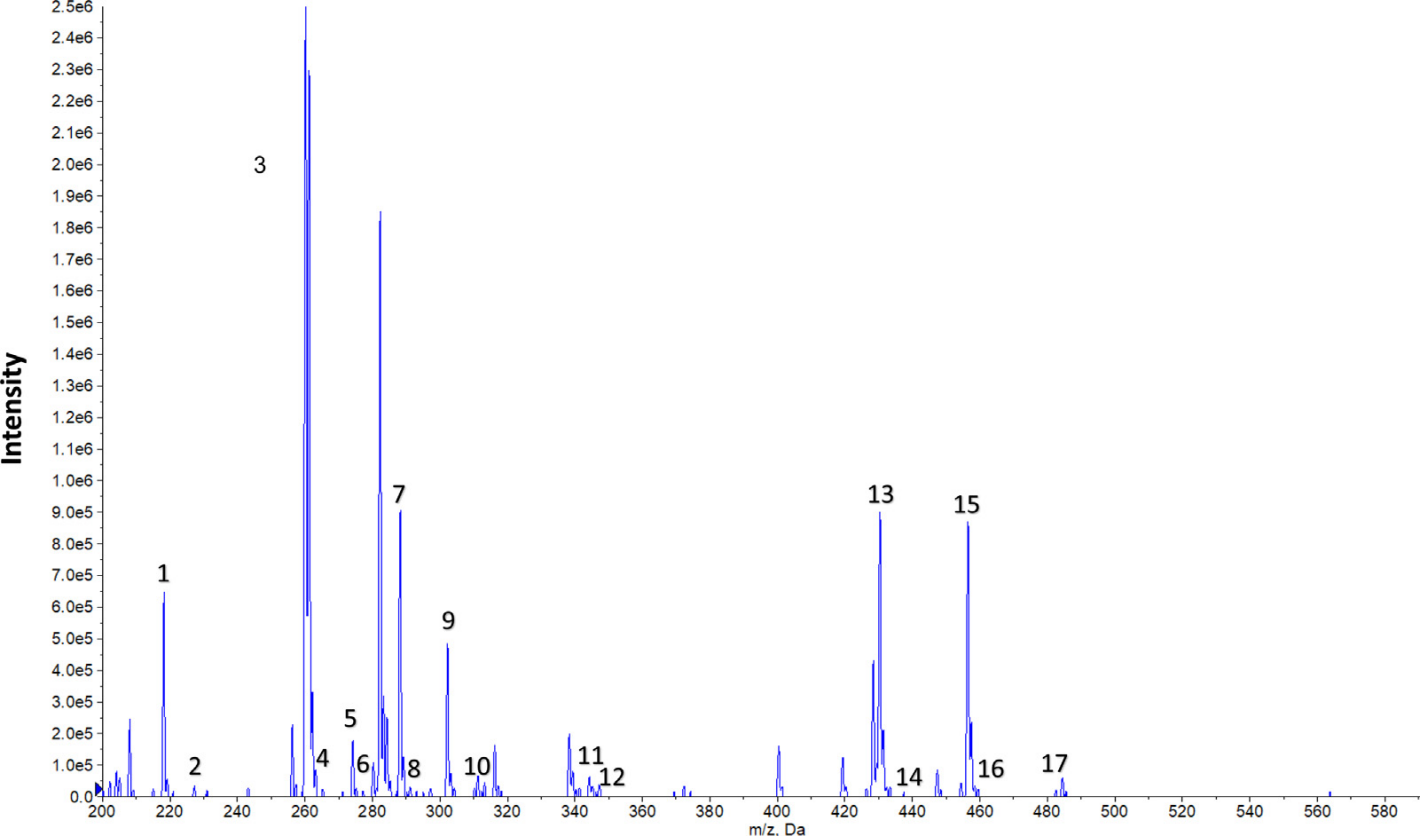
Acylcarnitines assay : represantative precursor ion scan (*m/z* 85) profile. (1) Free carnitine (C0), (2) C0 ISTD, (3) C2, (4) C2 ISTD, (5) C3, (6) C3 ISTD, (7)C4, (8)C4 ISTD, (9) C5, (10) C5 ISTD, (11) C8, (12) C8 ISTD, (13) C14, (14)C14 ISTD, (15) C16, (16) C16 ISTD, (17) C18.

### Small cellular metabolites by GC/MS profiling

#### Materials

Anhydrous pyridine (Sigma-Aldrich cat.no 270970)

Metoxyamine hydrochloride (Sigma-Aldrich cat.no 226904) prepare 20 mg/mL in anhydrous pyridine

*N*,*O*-Bis(trimethylsilyl)trifluoroacetamide/BSTFA (Sigma-Aldrich cat.no B-023)

#### Derivatization protocol

1. Dry combined polar and non-phases collected at step #11 /metabolism quenching and labeled as " *small cellular metabolites by GC/MS".*
2. Add 40 μL methoxyamine hydrochloride in pyridine (20 mg/mL).Cap tubes, vortex and incubate at 80 °C for 1 h.
3. Cool to the room temperature and add 60 μL N,O-Bis(trimethylsilyl)trifluoroacetamide (BSTFA). Cap tubes, vortex and incubate at 70 °C for 30 min.
4. Cool to the room temperature and transfer to vials for GCMS analysis.

#### GCMS parameters

**Table:** 

GCMS	
Ion Source	EI (Electron Ionization)
Source Temperature	280 °C
Quad Temperature	150 °C
Fixed Electron Energy	70eV
Acquisition Type	Scan
Injection Volume	1 uL
Solvent Delay	6 min
Column	HP-5MS 5% Phenyl Methyl Silox (30 m × 250 um × 0.25um)
Mode	Splitless

**Table:** 

Time Program	
Initial Setpoint	80 °C (hold 3 min)
Ramp	15 °C/min
Hold	305 °C (3 min)

##### Data analysis

Mass spectra analyzed by freely available AMDIS software and Fiehn library (Agilent). Identified metabolites are shown in Fig. 5. Normalize metabolite's levels to the reference standard tricarballylic acid *m/z* 377 (as BSTFA derivative) as following: use mass spectra to calculate the ratio of peak areas of target metabolites to the reference internal standard (tricarballylic acid) and to the total protein amount of each cell pellet as determined by the Bradford assay.

$$\text{Relative}\;\text{level}=\frac{\left[\text{Peak}\;\text{area}\;\text{of}\;\text{metabolite/}\left(\text{Peak}\;\text{ area}\;\text{of}\;\text{ reference}\;\text{standard}\right)\right]*\text{ reference}\;\text{standard}\;\text{ in}\;\left[\text{nmol}\right]\;\text{units}}{\text{Protein}\;\text{amount}}$$

#### Representative data

**Fig. 5:**
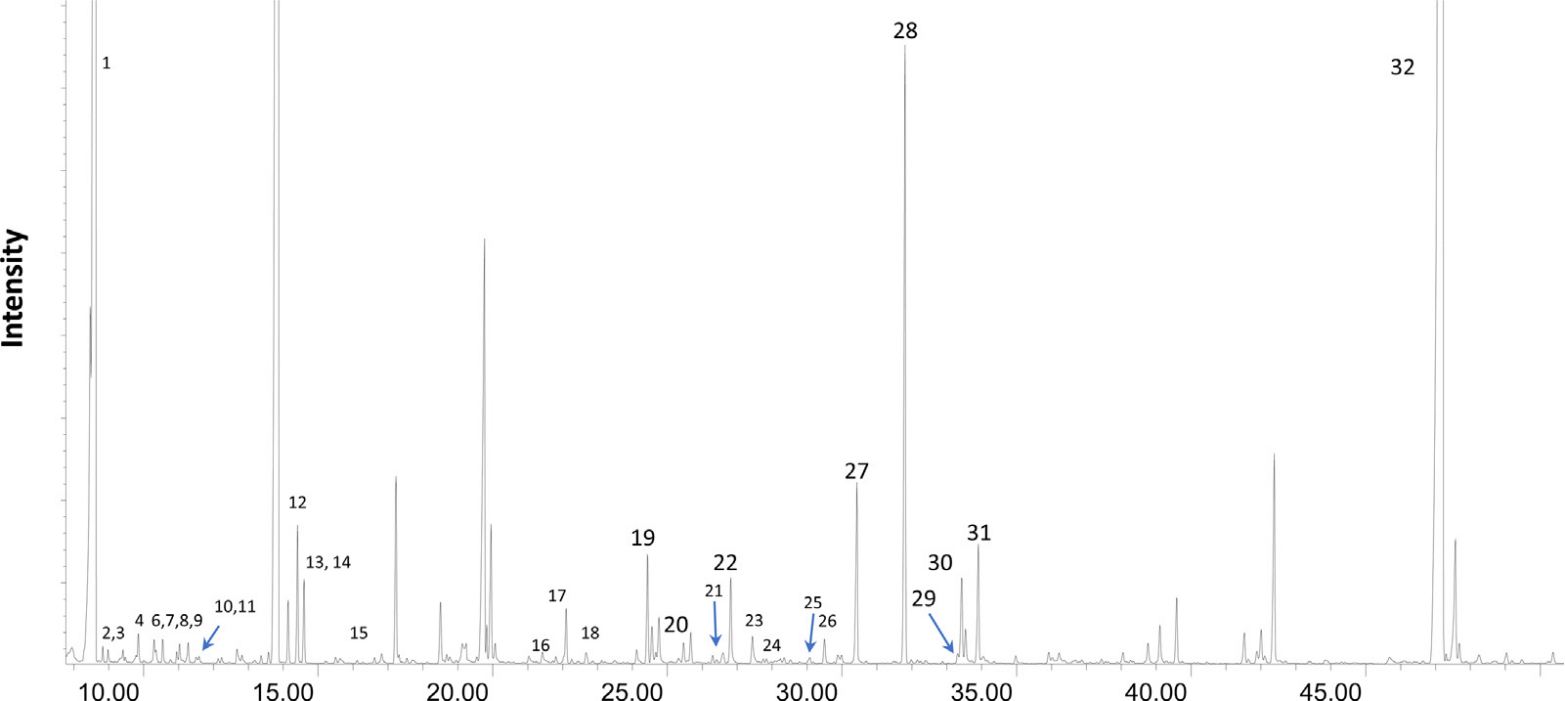
(1) Lactate, (2) Glycolic acid, (3) valine, (4) alanine, (5) leucine, (6)3-hydroxybutyric acid, (7)proline, (8) isoleucine, (9) serine, (10) octanoic acid, (11) glycine, (12)succinate, (13) fumarate, (14) threonine, (15) methionine, (16) aspartic acid, (17) malate, (18) glutamic acid, (19) glycerol-1-phosphate, (20) ornithine, (21) isocitrate (22) citrate, (23) tyrosine, (24) lysine, (25) ascorbic acid, (26) palmitoleic acid (27) palmitic acid, (28) inositol, (29) linoleic acid (30) oleic acid, (31) stearic acid, (32) cholesterol.

#### Glucose uptake, lactate and pyruvate production analysis

Glucose and lactate represent a major carbon sources for the myocardial energy metabolism. Whereas pyruvate is produced from lactate through the reaction catalyzed by lactate dehydrogenase, lactate to pyruvate ratio is a good surrogate for cytosolic redox state and correlates well with NAD^+^/NADH ratio.

For glucose uptake and lactate/pyruvate production assays incubate cells with ^13^C labeled glucose. We recommend to use a fully labeled commercial available ^13^C_6_ glucose.^13^C labeled glucose concentration in the cell media can be ranged from low (5 mM) to high (25 mM) concentration. Glucose uptake is measured by monitoring decrease in ^13^C_6_ Glucose concentration in culture media over the time (Fig. 8) and lactate and pyruvate production by the appearance of ^13^C_3_ labeled lactate (Fig. 8) and ^13^C_3_

#### Materials

^13^C_6_ labeled glucose (Sigma-Aldrich, cat.no 389374)

1 mM galactose (Sigma-Aldrich, cat.no PHR1206)

Tricarballylic acid (Sigma-Aldrich cat.no T53503) prepare 1 mM in water

Acetic anhydride (Sigma-Aldrich, cat.no 242845-5G)

Hydroxylamine hydrochloride (Sigma-Aldrich cat.no HX0770) prepare 0.2 M in anhydrous pyridine

Metoxyamine hydrochloride (Sigma-Aldrich cat.no 226904) prepare 20 mg/mL in anhydrous pyridine

*N*-*tert*-Butyldimethylsilyl-*N*-methyltrifluoroacetamide with 1% *tert*-Butyldimethylchlorosilane/ MTBSTFA (Sigma-Aldrich cat.no 375934)

Methanol (Sigma-Aldrich, cat.no 34860-1L-R)

Chloroform (Sigma-Aldrich, cat.no CX1050-1)

#### Protocol

1. Grow cells under standard culture conditions. To begin experiment with ^13^C_6_ glucose, aspirate media and wash cells twice with 1x D-PBS.
2. Add 2 mL of RPMI 1640 (no glucose) media supplemented with 5 mM–25 mM ^13^C_6_ labeled glucose.
3. In the course of the experiment, remove aliquots of cell media at 0, 30, 60, 120, and 240 min. Note-use more media initially if you plan to perform experiment for more than six hours.

#### Derivatization for GCMS analysis for the glucose uptake

1. To the 75 μL of the collected cell media aliquots add 25 μL of 1 mM galactose solution (used as internal standard) and 400 μL of methanol followed by 400 μL of chloroform.
2. Vortex samples and centrifuge at 5000 rpm for 8 min. Transfer upper polar phase to the new tube and dry under nitrogen at room temperature.
3. Add 100 μL of 0.2 M hydroxylamine hydrochloride in pyridine, cap tubes and incubate at 90 °C for 40 min.
4. Cool to the room temperature and then add 100 μL of acetic anhydride followed by incubation at 90 °C for 60 min.
5. Dry down derivatized samples by nitrogen stream in room temperature and re-suspend in 100 μL of ethyl acetate.

##### GCMS parameters

Following ions were monitored: *m/z* 319 for glucose, and *m/z* 314 for galactose.

**Table:** 

GCMS	
Ion Source	EI (Electron Ionization)
Source Temperature	280 °C
Acquisition Type	SIM
Injection Volume	1 uL
Solvent Delay	6 min
Column	HP-5MS 5% Phenyl Methyl Silox (30 m × 250 μm × 0.25 mm)
Mode	Splitless

**Table:** 

Time Program	
Initial set point	80 °C (hold 3 min)
Ramp	15 °C/min up to 180 °C
Ramp	5 °C/min up to 205 °C
Ramp	1 °C/min up to 212 °C
Ramp	15 °C/min up to 310 °C min
Hold	310 °C (5 min)

#### Representative chromatogram

**Fig. 6:**
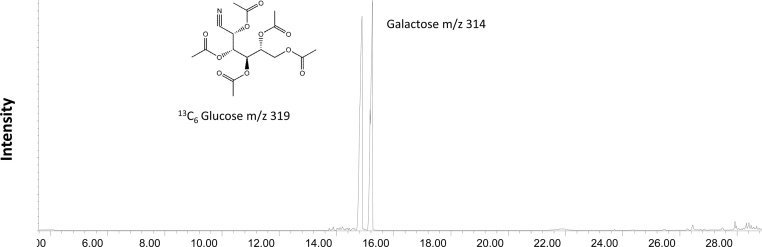
Representative GCMS chromatogram. Cell media extract ^13^C_6_ Glucose and galactose as internal standard.

#### Derivatization protocol for the lactate and pyruvateproduction assay

1. To the 75 μL of the collected cell media aliquots (Step #3) add 25 μL of 1 mM tricarballylic acid and dry samples.
2. Add 40 μL methoxyamine hydrochloride in pyridine (20 mg/mL), vortex and incubate at 80 °C for 1 h.
3. Cool to room temperature and add 60 μL MTBSTFA. Cap tubes, vortex and heat at 70 °C for 30 min.
4. Cool to the room temperature and transfer to GCMS vials.

#### GCMS parameters

Following ions were monitored: for ^13^C_3_ lactate *m/z* 264, for ^13^C_3_ pyruvate *m/z* 177 and *m/z* 377 for tricarballylic acid.

#### Data analysis

Note: Data analysis was performed under validated assumption that there is no significant natural abundance contribution to the ^13^C_3_-Lactate and ^13^C_3_pyruvate, thus for the concentration calculations the data was not corrected for ^13^C natural abundance.

Relative level=[Peak area of C313l lactate/(Peak area of referencestandard)]*reference standard in [nmol] unitsProtein amount

Relative level=[Peak area of C313 pyruvate/(Peak area of reference standard)]*reference standard in [nmol] unitsProtein amount

#### Representative chromatogram

See Fig. 7.

**Fig. 7:**
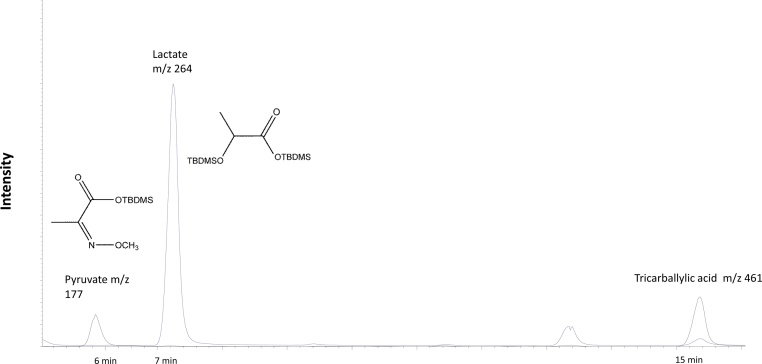
Representative GCMS chromatogram. Cell media analysis for pyruvate and lactate.

**Fig. 8:**
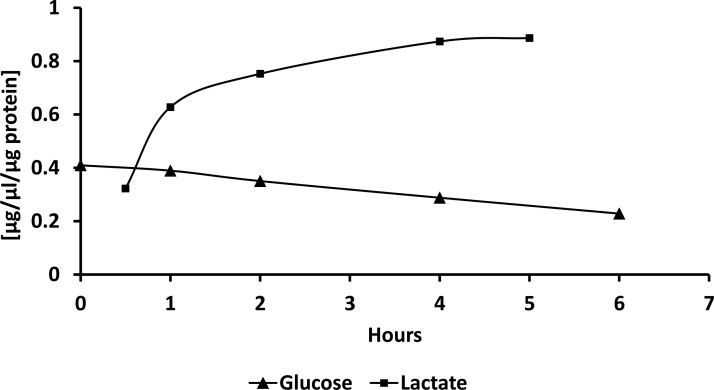
Representative ^13^C_6_ glucose uptake and ^13^C_3_ lactate production for iPSCM.

## Results and discussion

In recent years there are emerging evidence for studies involved human iPSCM in basic science, regenerative medicine, and the pharmaceutical industry. Although much progress has been made in the genetic and electrophysiological characterization of iPSCMs, it is not understood well how metabolic phenotypes are affected in the course of the disease or in response to newly developed therapies. Thus, to fulfill the potential for iPSCM related studies, there is a need for metabolic assays complementary to the existing functional characterization methods. Here, we developed mass spectrometry-based quantitative assays and applied them for metabolic characterization of iPS-CM. Using these assays we successfully profiled a variety of small metabolites involved in different biochemical pathways.

Acylcarnitines have a central role in the transport of fatty acids into the mitochondria for subsequent β-oxidation. Acylcarnitine analysis can be especially useful for iPSCM models of heart failure [5,6], diabetic cardiomyopathy [7] and fatty acid oxidation disorders (FAOD) with cardiac manifestation [8,9]. Given the fact that the relative acylcarnitines pools and individual contributions within the total carnitine pool are closely correlated to the intra-mitochondrial acyl-CoA pools it is suggested that proposed assay is a powerful tool for the mitochondrial dysfunction investigations in iPSCM. Amino acids also have a high significance to heart metabolism. Although under physiological condition, the human heart has minimal reliance on amino acids for ATP, some amino acids replenish citric acid cycle and their utilization increases during heart failure, hypoxia or another pathological metabolic remodeling. The proposed high throughput assay analyses acylcarnitines and amino acids in two minutes and has an automated data analysis algorithm (Chemoview/ABSCIEX) but can be also performed manually.

### GCMS metabolic profiling

The proposed gas chromatography-mass spectrometry metabolic profiling allows semiquantitative analysis of small metabolites (50–650 Da). Although method requires extensive sample preparation including chemical derivatization [10] to increase metabolites' volatility for gas chromatography, it was able to detect thirty two metabolites extracted from one well (standard six well plates, 1.2–1.5 * 10^6^ iPS-CM per well in average). For metabolites identification we applied freely available deconvolution software AMDIS [11] and commercially available spectral library [12]. Under standardized EI-GCMS conditions, every metabolite produced unique fragmentation pattern that was matched to the library based on >78% match criteria. The identified small metabolites included amino acids, carbohydrates, fatty acids, and sterols.

### Lactate/pyruvate assay

Pyruvate is the end product of glycolysis and it also can be produced from lactate through the reaction catalyzed by lactate dehydrogenase. Thus, lactate to pyruvate ratio is a good surrogate for cytosolic redox state and correlates well with NAD+/NADH ratio. Dysfunctions in a citric acid cycle or respiratory chain may lead to reduced pyruvate oxidation and to the abnormal lactate to pyruvate ratios. At the same time, excessive lactate production is an indication of lactic acidosis which is detrimental to the iPSCM contractility.

### Glucose uptake assay

In iPSCM cellular system, glucose is a primary energy source, thus uptake assay is an important tool. Alterations in glucose uptake can reflect overall metabolic changes, and in conjunction with the pyruvate production can indicate glycolysis flux changes. At the same time, glucose uptake monitoring can be indicative of the over or down expression of glucose transporters.

## Conclusion

We have developed a workflow for the analysis of small metabolites in iPSCM involved in energy-related pathways. The workflow combines GCMS and LC–MS/MS platforms serves as an important tool for iPSCM metabolic characterization and can provide complementary metabolic endpoints to monitor therapeutic interventions. Future studies to expand iPSCM metabolic profiles are warranted.
